# 1.S. Preparing for the unexpected

**DOI:** 10.1093/eurpub/ckac130.014

**Published:** 2022-10-25

## Abstract

This abstract has been withdrawn

